# Detection of myocardial inflammation in Chagas' disease by cardiac magnetic resonance

**DOI:** 10.1186/1532-429X-15-S1-M12

**Published:** 2013-01-30

**Authors:** Jorge A Torreão, Evandro Naia, Carlos H Rassi, José R Parga, Luis F Ávila, Cesar H Nomura, Barbara M Ianni, Charles Mady, Roberto Kalil-Filho, Carlos E Rochitte

**Affiliations:** 1 Cardiology, Cardiovascular Magnetic Resonance Sector, Heart Institute, InCor, University of São Paulo Medical School, Sao Paulo, Brazil

## Background

Chagas disease (CD) is an important health problem in South America and, with increasing migration to North America and Europe, it is becoming a global challenge. The disease' pathogenesis is not fully understood, especially in its subclinical forms. We sought to investigate the presence of myocardial fibrosis, edema and hyperemia in three clinical forms of the disease.

## Methods

Fifty-four patients with CD were analyzed, 16 patients in the indeterminate form (IND), 17 patients with the cardiac form without systolic dysfunction (CF), and 21 patients with the cardiac form with systolic dysfunction (CFSD). All patients underwent 1.5-T cardiac magnetic resonance (CMR) using three pulse sequences, previously described as useful for the diagnosis of viral myocarditis: the myocardial delayed enhancement (MDE) technique, Triple-IR FSE T2-weighted sequence and the T1 weighted global enhancement acquired before and after contrast injection, to identify fibrosis, edema and hyperemia of the myocardium, respectively. The parameters for all sequences followed precisely the recommendations for acute myocarditis published on JACC White Paper (Friedrich et al. J Am Coll Cardiol. 2009 Apr 28;53(17):1475-87).

## Results

Myocardial fibrosis was present in 72.2% of all patients, in 12.5% of IF, 94.1% of the CF and 100% of the CFSD (p < 0.0001). Myocardial mass of fibrosis was 2.8g (2.8% of LV mass) in the group of 24 patients with ejection fraction over 55%, 19.6g (15.1%) in the group of 17 patients with the ejection fraction between 35% and 54.9% and 29.0g (21.5%) in the group of 12 patients with ejection fraction under 35% (p< 0.0001). Myocardial edema was present in 77.8% of all patients, in 31.3% of the IF, 94.1% of the CF and 100% of the CFSD (p < 0.0001). Myocardial edema extension was analyzed by number of segments: 0.2 segment in the group of 24 patients with ejection fraction over 55% (12 with positive edema), 2.0 segments in the group of 18 patients with the ejection fraction between 35% and 54.9% and 2.25 segments in the group of 12 patients with ejection fraction under 35% (p< 0.0001).

Myocardial hyperemia was present in 73.8% of all patients, in 25.0% of the IND, 92.3% of the CF and 94.1% of the CFSD (p < 0.0001). In the analysis by ejection fraction, hyperemia was observed in 47.3% of patients with ejection fraction over 55%, 93.3% of patients with the ejection fraction between 35% and 54,9% and 100% of patients with ejection fraction under 35% (p< 0.001).

## Conclusions

Inflammation of the myocardium can be detected by CMR on patients with CD, including in patients in the subclinical phase. Prevalence and magnitude of myocardial inflammation progressively increases with the more advanced forms of CD. Additionally, the presence and magnitude of MDE, myocardial edema and hyperemia were associated with deteriorated left ventricular systolic function.

## Funding

FAPESP 2010/51900-3

